# Preliminary Characterization of an Active CMOS Pad Detector for Tracking and Dosimetry in HDR Brachytherapy

**DOI:** 10.3390/s24020692

**Published:** 2024-01-22

**Authors:** Thi Ngoc Hang Bui, Matthew Large, Joel Poder, Joseph Bucci, Edoardo Bianco, Raffaele Aaron Giampaolo, Angelo Rivetti, Manuel Da Rocha Rolo, Zeljko Pastuovic, Thomas Corradino, Lucio Pancheri, Marco Petasecca

**Affiliations:** 1 Centre for Medical Radiation Physics, University of Wollongong, Wollongong, NSW 2522, Australia; tnhb570@uowmail.edu.au (T.N.H.B.); mlarge@uow.edu.au (M.L.); joel.poder@health.nsw.gov.au (J.P.); joseph.bucci@health.nsw.gov.au (J.B.); 2 St George Cancer Care Centre, Kogarah, NSW 2217, Australia; 3 School of Physics, University of Sydney, Camperdown, NSW 2050, Australia; 4 Department of Electronics and Telecommunications, Polytechnic University of Turin, 10129 Turin, Italy; ebianco@to.infn.it (E.B.); raffaeleaaron.giampaolo@to.infn.it (R.A.G.); 5 Istituto Nazionale di Fisica Nucleare—Section of Turin, 10125 Turin, Italy; rivetti@to.infn.it (A.R.); darochar@to.infn.it (M.D.R.R.); 6 Australian Nuclear Science and Technology Organisation, Lucas Heights, NSW 2234, Australia; zkp@ansto.gov.au; 7 Department of Industrial Engineering, University of Trento, 38123 Trento, Italylucio.pancheri@unitn.it (L.P.); 8 Trento Institute for Fundamental Physics and Applications, Istituto Nazionale di Fisica Nucleare, 38123 Trento, Italy

**Keywords:** brachytherapy, CMOS, source tracking, active pixel, dosimetry, IBIC

## Abstract

We assessed the accuracy of a prototype radiation detector with a built in CMOS amplifier for use in dosimetry for high dose rate brachytherapy. The detectors were fabricated on two substrates of epitaxial high resistivity silicon. The radiation detection performance of prototypes has been tested by ion beam induced charge (IBIC) microscopy using a 5.5 MeV alpha particle microbeam. We also carried out the HDR Ir-192 radiation source tracking at different depths and angular dose dependence in a water equivalent phantom. The detectors show sensitivities spanning from (5.8 ± 0.021) × 10^−8^ to (3.6 ± 0.14) × 10^−8^ nC Gy^−1^ mCi^−1^ mm^−2^. The depth variation of the dose is within 5% with that calculated by TG-43. Higher discrepancies are recorded for 2 mm and 7 mm depths due to the scattering of secondary particles and the perturbation of the radiation field induced in the ceramic/golden package. Dwell positions and dwell time are reconstructed within ±1 mm and 20 ms, respectively. The prototype detectors provide an unprecedented sensitivity thanks to its monolithic amplification stage. Future investigation of this technology will include the optimisation of the packaging technique.

## 1. Introduction

Brachytherapy and in particular high dose rate brachytherapy (HDR-BT) is a common treatment modality for prostate cancers with extensively documented positive clinical outcomes [1]. In Australia, it was estimated that 3152 deaths have occurred due to prostate cancer in 2020 (Australian Government—Cancer Australia 2020). HDR-BT has been a safe and an effective treatment modality, but poor execution, miscalculation and possible technologically related errors have been documented resulting in a high variation between the planned and delivered dose distributions. There are published documents on errors in brachytherapy and their potential health risk, highlighting that the negative impact of small deviations from the irradiation treatment plan generated by misplacement of the catheters or sequence errors can lead to significant consequences and affect patient outcomes if not properly handled [2,3]. Improving treatment verification and providing an in-vivo quality assurance (QA) method can lead to optimal patientall outcome. The recommendation from the American Associations of Physicists in Medicine (AAPM) Radiation Therapy Task Group (TG) No. 59 [4] and AAPM Report from the TG-56 [5] suggested that a regular basis check on the source dwell time, position and transit time minimise potential errors during the treatment process [6,7,8].

According to Ref. [6], the goals of in-vivo dosimetry include providing the clinicians with a relevant assessment of the quality of brachytherapy treatment progression to enable prompt intervention in case of clinically relevant errors. Traditionally, this has been carried out by measuring the dose in specific accessible points within the human body (rectum, urethra, cervix, oral cavity). Such detectors should be able to resolve the motion of the source between dwell positions in increments of 1 mm, with a minimum dwell time of 100 ms (used by the treatment planning system to calculate the dose) in a range of distance from 5 to 40 mm (in the case of HDR brachytherapy of prostate cancer). Their response should be linear with incremental dose, dose rate and should be angular independent and been able to be located precisely in respect to the patient anatomy. In-vivo dosimetry would be more effective if it is also time-resolved. So, the detector should have an instantaneous response (sensor response is proportional to dose rate) which would allow to be used for source tracking but also for real-time interruption and correction (adaptive in-vivo dosimetry) of the treatment.

Several research studies have shown promising results and potential clinical impact for use of in-vivo source tracking for prostate [9,10,11,12,13,14], gyno [15] and general HDR applications [16,17,18,19]. Some of these studies have adopted silicon diodes, in the form of single devices, linear or 2D arrays showing positive results in source tracking [15,20]. The work in [11] in particular, adopts a 2D array of 121 silicon diodes with no bias and readout by a multichannel electrometer named “Magicplate”. This study, along with others, has successfully developed a reconstruction technique for positional and timing estimation of the HDR source in 3D. Its applicability for direct dose measurement is limited since the sensor is not positioned in proximity of the target volume and so it can estimate the dose only by reconstructing its dwell position and time. The limitation of a standard silicon diode technology arises from the challenge of building a solid-state sensor with high sensitivity, limited energy dependent response and a wide dynamic range. An electronic analogue front-end circuit with high gain is required to readout a silicon diode but, these studies showed that also noise issues due to electromagnetic interferences occurred. This problem originates from the large physical distance between the sensor (which is supposed to be placed inside the body or in close proximity of the patient) and the electronic front-end. The mechanical support required for the detector also contains the connections of the sensors to the electronic readout, creating a parasitic antenna able to pick up the radiofrequency noise created by the HDR afterloader and any other electronic device in the treatment theatre.

A different approach that overcomes this problem is to use a plastic scintillator, which flashes visible light when interacting with radiation. Light can be transferred directly to the electronic readout by a fibre optic cable. This technique is named Fiber Optic Dosimetry and has been proposed and developed by Beddar et al., to measure dose directly in the urethra or rectum [21]. The energy deposited in the plastic scintillator is readout by a photomultiplier tube, which is several meters away from the source of radiation. The limitation of this technique is the presence of a parasitic optical signal from Cerenkov light generated by ionising radiation directly into the fibre optic, affecting the linearity and accuracy of the sensor with accumulated dose. The subtraction of such signal is possible by several techniques but the most reliable is to use two optic fibres, one with the scintillator and another one without it [22]. This solution makes multi-point dose measurement challenging due to the number of channels required and complexity of the readout electronics.

The aim of this study is to investigate the use of a microelectronic semiconductor processing to fabricate a silicon radiation detector for dosimetry with an embedded electronic readout based on standard complementary metal-oxide-semiconductor (CMOS) technology. This kind of detector belongs to the family of monolithic active pixels, developed primarily for imaging and high-density, high-resolution radiation detection applications. Being a sort of “camera on chip”, active pixel sensors integrate the electronic amplifier and have shown high potential and to be beneficial in the applications for high resolution imaging [23,24,25] and for radiation particle tracking [26,27,28,29,30]. An attempt to adopt such CMOS imaging devices for dosimetry has been proposed previously by [31] and also by [32]. They both adopted a CMOS imaging sensor (2D array of active pixels with an embedded source-follower amplifier), originally developed for ionizing radiation imaging. They used it to track and detect the intensity of brachytherapy sources in plastic phantoms. In [31], authors describe a custom designed sensor with more than 300,000 pixels and an overall size of 14 × 14 mm^2^. The sensor is readout at a frequency of 5 Hz or every 200 ms, which is one order of magnitude too slow to resolve the transient time of the source or two times too slow to identify short dwell positions of the HDR source (minimum dwell time is 100 ms). While this technique provides a sensitive method to detect deviations from the treatment plan during delivery, it does show three major limitations: 1. The clinical significance of any detected changes is given by the effective dose variations which cannot be estimated using this type of imaging sensors; 2. As a result of the number of pixels of the imager, its time response is too slow to resolve the fast transients of the HDR afterloader or short dwell positions as required by the treatment planning system. 3. The size of the sensor which is way too large to be inserted inside the patient. Ref. [32] suggests the use of a commercial CMOS flat panel and the concept is proved using low dose rate preloaded brachytherapy needles. The technique they suggest is able to reconstruct the position of the sources accurately but it lacks of temporal resolution since the sensor adopted is designed to integrate the signal generated by radiation on long time intervals (longer than 100 ms as required in HDR bachytherapy) and do not provide direct measurement of the dose delivered by the sources.

The approach proposed in this work takes advantage of the accuracy and reproducibility of CMOS radiation sensors and embedded electronic readout but overcoming the limitations of low time resolution and capability of direct dose measurement. We achieved this objective by re-arranging the architecture of the sensor and optimizing the electronic readout by a designed-on-purpose preamplification stage.

## 2. Materials and Methods

### 2.1. Detector Architecture

**Figure 1:**
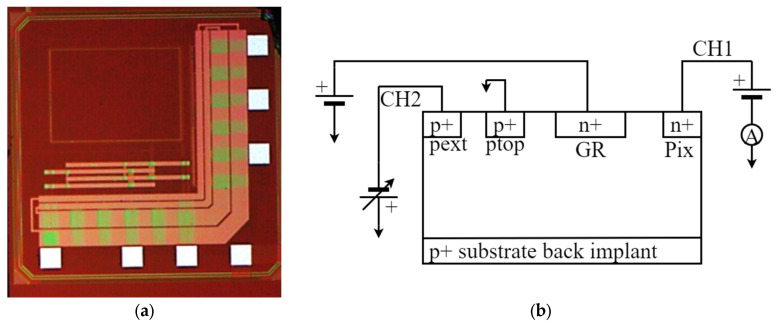
(**a**) Detector top view: the detector is the light red square in the top-left corner. The CMOS preamplifiers are the pink areas on the right hand and bottom side of the chip; (**b**) Schematic representation of the diode structure and the external connections used to measured current-voltage characteristics.

The new prototype detector is a combination of an active silicon pad detector (600 μm × 400 μm) connected to a built-in CMOS electronic front-end consisting of two alternative amplification stages and one line driver (buffer). For the purpose of characterization, the detector has been encased in a ceramic package as shown in Figure 1a.

The architecture of the detector is illustrated in Figure 1. The detector is a silicon high resistivity p-i-n device (Figure 1b). Two different wafers have been used to fabricate the same topology and are identified as Wafer10 and Wafer20, for the 100 μm and 48 μm thick active substrate, respectively. The detector’s p-n junction is biased through an external p-well (coupled by a low impedance path to the back junction), and by the n+ pad junction on the top side, surrounded by p-wells to form a multi-stage guard ring block. Wafer20 is 48 μm thick of high resistivity substrate built on a 250 μm handling wafer of very low resistivity, which forms also the back ohmic contact. Details related to resistivity of the substrates, structure of the guard-rings and doping concentrations are commercial in confidence and cannot be disclosed.

### 2.2. Active Pad Architecture and Probe Assembling

The detector is a monolithic active pad device fabricated on a standard deep sub-micron 110-nm CMOS technology [33]. The detector is integrated in the same substrate with a dual analogue frontend configuration: a charge sensitive amplifier (CSA) which allows the collection of the current generated in the pad by ionising radiation and converts it into a voltage signal proportional to the charge integrated in a defined time frame. The second topology adopts a transimpedance amplifier (TIA) which provides a voltage signal proportional to the current. The detector readout modality can be selected by a set of 3 bits. The CSA is designed specifically for low dose rate brachytherapy to perform spectroscopical dosimetry as reported in other works [34]. In this work, we have considered only the TIA modality to readout the detector due to the higher intensity of the dose rate and the wider spectrum of the source adopted in HDR brachytherapy.

**Figure 2:**
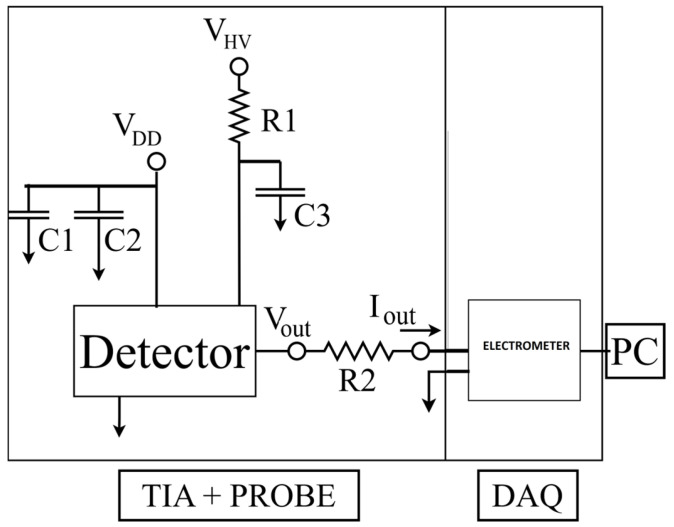
TIA and Probe circuit.

The sensor requires the connection for the bias of the detector (V_HV_) with a bypass filter for noise rejection. The electronics is biased by +3.3 V (V_DD_) and a reference common ground (Figure 2). The voltage output signal (V_OUT_) formed by the electronic frontend of the chip is readout by a custom designed Data Acquisition System (DAQ) through a 1 MΩ resistor (R2) and digitized by the DDC264 Texas Instrument multichannel electrometer. The digital data are packaged and transferred to a standard laptop through a USB2.0 protocol, managed by a custom designed graphical interface. For a detailed description of the electrometer and data acquisition system, please refer to [35]. Converting the sensor’s voltage output signal through the resistor R2 is required since the DAQ is optimised for current signal readout. The resistor has been placed very close to the input of the electrometer to minimise the noise due to radiofrequency interferences.

### 2.3. Phantom

**Figure 3:**
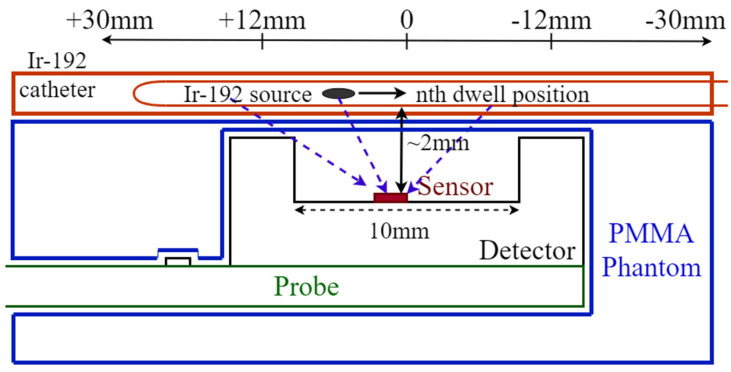
Schematic of the sensor assembled on the probe and placed in the phantom along with the relative position of the Ir-192 catheter (diagram is not to scale).

**Figure 4:**
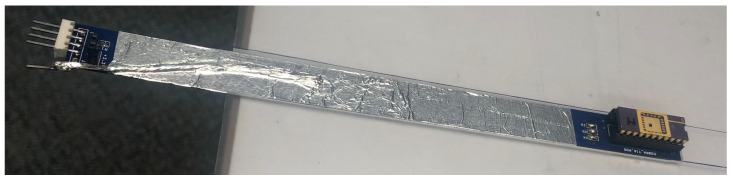
Probe assembled and placed above the PMMA phantom.

In order to protect the sensor from mechanical impacts during measurements and provide a water equivalent wall for the dosimeter, a stack of two PMMA slabs of 30 × 30 cm^2^ are adopted to encase it. The top slab is 10 mm thick and a cavity is machined at the centre (30 mm × 16.5 mm × 9 mm) to make space for the probe, allowing the sensor to face only 2 mm of PMMA (Figure 3) above it. A slab of 5 mm is placed below the detector to provide adequate backscattering, minimising airgaps and seal the encasing for protection of the sensor. The HDR brachytherapy catheters are encased in a 5 mm thick PMMA slab, placed immediately above the sensor slab, creating a minimum water equivalent distance of the catheter from the sensor of 2 mm (Figure 3). The pad detector is mounted on a probe formed by a 210 mm × 16 mm printed circuit board along with a connector to allow the communication with an external electrometer (Figure 4).

### 2.4. Electrical Characterization

A current−voltage (IV) characteristic in biased silicon detectors provides an insight in the quality of the fabrication and the expected baseline to be subtracted from the signal. The detector proposed in this work is a monolithic device with the sensor embedded in the same substrate with the electronic readout. This architecture makes it inconvenient to measure the current-voltage characteristics (IV). In order to measure the sensor IV, a series of bare pad test structures have been fabricated in the same substrate. A voltage sweep from 0 V to −40 V using a Keithley 230 Programmable Voltage Source has been carried out following the schematic connections shown in Figure 1b. Separate fixed input biases (1.2 V, 2 V and 3 V) from a battery pack were applied to the guard ring (G.R) to isolate the pad detector and minimise surface leakage currents. The Ptop junction was grounded to avoid a direct forward potential across the P+ back junction and the guard ring. This is the same bias configuration provided by the electronic readout to the pad detector in the monolithic sensor, and it has been replicated to provide consistent bias conditions as for the detector during normal operation. We have also performed a test on an un-diced detector (detector fabricated in the same batch of production but still on the wafer). It was important to establish the effects of the cutting process on the leakage currents and on the signal baseline.

### 2.5. Charge Collection Imaging by Ion Beam Induced Charge (IBIC) Microscopy 

**Figure 5:**
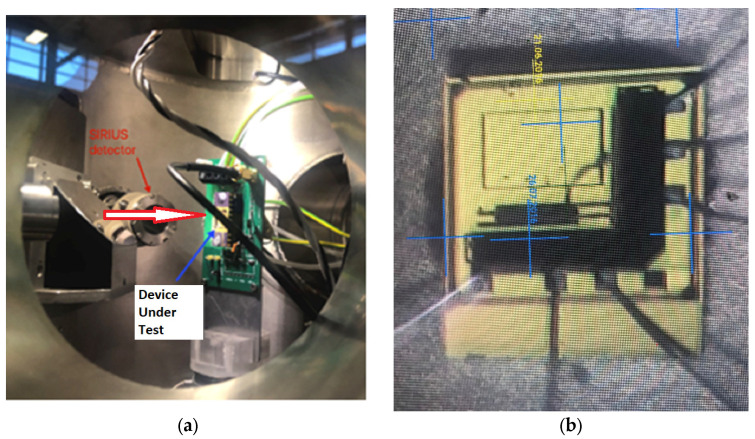
(**a**) View of the sensor placed inside the vacuum chamber of the accelerator at ANSTO; the arrow describes the direction of the beam incident on the sample (**b**) beam view of the detector under the microscope.

Charge collection imaging of an operating prototype detector by IBIC microscopy [36] is twofold critical. It provides the size and uniformity of the sensitive volume and an estimation of the signals (transient pulses) or signal distortions due to the effects of radiation interacting with the electronic front−end. The latter is also crucial in order to establish potential distortions created by single and cumulative radiation effects. The IBIC microscopy measurements have been performed using the SIRIUS heavy−ion microprobe at the Centre for Accelerator Science of the Australian Nuclear Science and Technology Organisation (ANSTO) [37,38]. The detector was irradiated with a rapid raster-scanning microbeam of 5.5 MeV alpha particles focused down to a 1 μm spot size, with the particle event rate between 800–1000 Hz and the scanning area of 1 × 1 mm^2^ (Figure 5). 

The sensor’s response was calibrated by using the pristine alpha particle energy deposition and the residual energy after two different layers of absorbing materials: 13 μm of polyimide (Kapton) and, alternatively, 6 μm of Mylar. The Constant Slowing Down Approximation range (CSDA) for 5.5 MeV Alpha was estimated from the National Institute of Standards and Technologies (NIST) database for all of the materials [39]. For silicon, a range of 27.85 μm is expected for the total incident alpha particle energy. Assuming full absorption in substrates 100 μm and 48 μm thick, a conversion factor expressed in units of Channels/MeV was obtained. The IBIC results are Median Energy Maps (MEM) and Median Energy Windowed Maps (MEWM) were obtained for both Wafer10 and Wafer20 fabrication options of the samples. A median map is obtained correlating each current pulse amplitude (proportional to the energy deposited by the particle) with the impact position on the surface of the sensor. The position is obtained knowing the steering angle of the beam steering magnets relative to the sensor position in the vacuum chamber. For further details on the methodology for charge collection mapping developed at ANSTO, see [37,38].

### 2.6. Characterization of the Detector Response to an Ir-192 Gamma Source for HDR Brachytherapy

The aim of this set of measurements carried out at St. George Hospital, Kogarah, Australia, is a preliminary evaluation of the performance of the active pad sensor in detecting a HDR brachytherapy source in a plastic phantom for tracking and direct dosimetry.

In order to achieve this purpose, an HDR afterloader equipped with an Iridium192 (Ir-192) source was adopted. The source, an Ir-192 Nucletron Flexisource (Elekta, Veenendaal, The Netherlands), had an activity of 252.9 GBq, Air Kerma Strength 27.89 mGy m^2^ h^−1^; the afterloader has a nominal positional error of ±0.2 mm, minimal step size of 1 mm and 0.1 s minimal dwell time [40,41].

#### 2.6.1. Source Localization and Tracking

Tracking of the radioactive source while it is dwelling horizontally in an implanted catheter, requires: the ability to detect the radiation emitted by the source, evaluate the distance based on the response of the sensor and the activity of the source, record the time at which the source is dwelling and the time at which it is travelling between two dwell positions. In order to test the performance of the sensor in tracking the Ir-192 source, the detector was positioned below the PMMA slab with a recess to accommodate the HDR catheters as shown in Figure 3. The tip of the catheter was placed at 30 ± 0.1 mm from the sensor and at a vertical distance of 2 mm. A plan has been designed to dwell the source in 21 positions, along a total distance of 60 mm from the tip of the catheter, in 3 mm increments and 1 s dwell time at each position. 

A bias of −31.64 ± 0.10 V was supplied to the detector throughout the tracking experiment. Due to the high sensitivity of the electrometer, the bias of the sensor had to be adjusted to avoid saturation of the readout in other experiments and it will be mentioned if different. 

#### 2.6.2. Sensitivity

The activity of the Ir-192 source (half-life is 74 days) can be considered constant during the time of a treatment, so the total dose deposited in a point depends on the distance of the source from the detector and the dwell time. In order to measure the sensitivity, the source is dwelled above the sensor in a catheter embedded in the PMMA phantom at a depth of 7 mm and dwell time increased accordingly with the expected dose calculated by the Treatment Planning System (Oncentra Brachy version 4.5.3, Elekta, Veenendaal, The Netherlands). The dose accumulated in the same conditions used for the sensitivity were simulated using the TPS (Treatment Planning System) for a total dose ranging from 0.5 to 5.2 Gy, respectively. TPS calculates the dose in the hypothesis that all space surrounding the source is water. A detailed description of the dose calculation for Ir-192 Flexisource can be found in [40] and AAPM TG−43 Protocol [41].

#### 2.6.3. Depth Dose

The measurement of the dose depth distribution in a water equivalent material is a test to evaluate the effective tissue equivalency of the response of the sensor to the Ir-192 radiation field. The depth dose has been measured by dwelling the source for 1 s at different distances above the sensor, from a minimum of 2 mm to a maximum distance of 52 mm. The conversion of the detector response in absorbed dose (nC/Gy) in water is obtained from the sensitivity measurement and applied to the variation of the response with depth. In order to benchmark the accuracy of the dose measured by the sensor, the data have been compared to the calculation based on the TG-43 protocol embedded in the TPS.

The detector response as a function of depth also provides a good estimation of the sensitivity of the sensor in detecting the faint signal generated by the decreasing flux of the radiation emitted by Ir-192 in water. Reproducibility and uncertainties have been calculated as one standard deviation of three measurements for each depth.

## 3. Results

**Figure 6:**
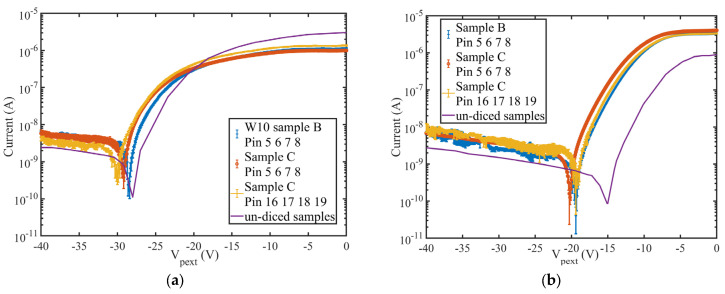
Current-Voltage characteristics of the samples fabricated on Wafer10; (**a**) for a biasing of the pixel (VPIX) with an offset of 1.202 V, (**b**) with an offset of 2.02 V. The solid lines represent the IV from the un-diced samples.

Figure 6 shows the leakage current as a function of the voltage applied to the backside p+ contact. The non-zero current recorded at 0 Volt, is due to the presence of a positive voltage at the pad junction equal to 1.202 V (Figure 6a). This voltage (Pix in Figure 1b) represents the input offset generated by the electronics connected at the pad detector in the final sensor architecture. In order to have an accurate estimation of the leakage current in operating conditions, we have set the same bias conditions of the sensor as expected in the final architecture. Since a virtual zero voltage is applied to the junction P_ext, the diode is forward biased and produces a large current as expected. When the potential of the backside is negatively increased, the depletion generated in the substrate, produces an isolation between the standard guard ring, kept to a slightly positive voltage of 1.00 ± 0.01 V, and the pad junction. It is clear from the IV plots, that a minimum voltage of −29 ± 0.1 V must be applied in order to produce a depletion that sets the guard ring effectively and minimises the leakage current. Figure 6a shows the IV plots of three samples: sample B and C (blue and red symbols) are samples tested on the wafer while sample C with the yellow symbol is the same detector after the dicing.

Each “sample” contains 4 different diodes (identified by their pin number); they have been measured individually and their current averaged for each voltage applied. The error bars show two standard deviations for all of the measurements.

Figure 6b shows the leakage current if an offset of 2.02 ± 0.01 V is applied to the pad detector. The offset greatly reduces the depletion voltage required but increases the minimum leakage current obtained. For the samples fabricated on Wafer10, the depletion voltage threshold varies from −29 V to −20 V but the current increases by almost an order of magnitude, making the bias of the pad detector with 1.202 V the preferred choice. This finding is in substantial agreement with the results obtained on the tests performed on the uncut wafer samples [33]. The depletion threshold of the cut samples increases of approximately 2 ± 0.1 V in respect to the un-cut samples, corresponding approximately to a variation between 10 to 20% of the leakage current.

### 3.1. Charge Collection Mapping (IBIC)

**Figure 7:**
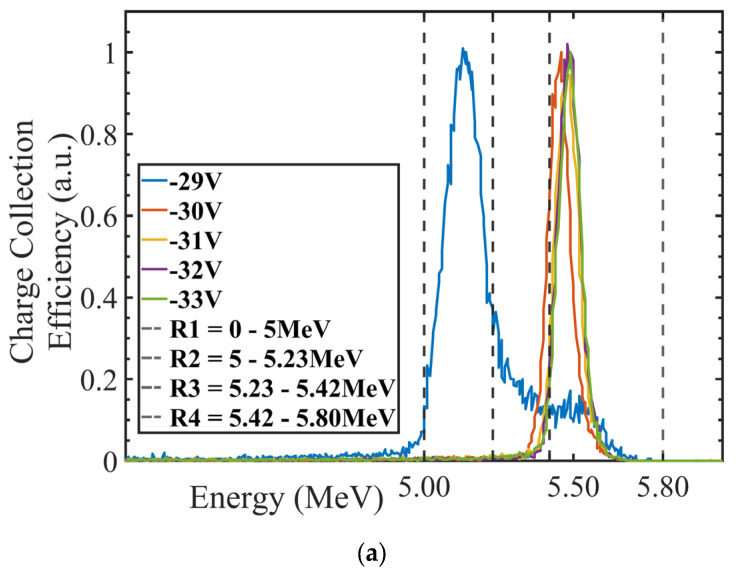

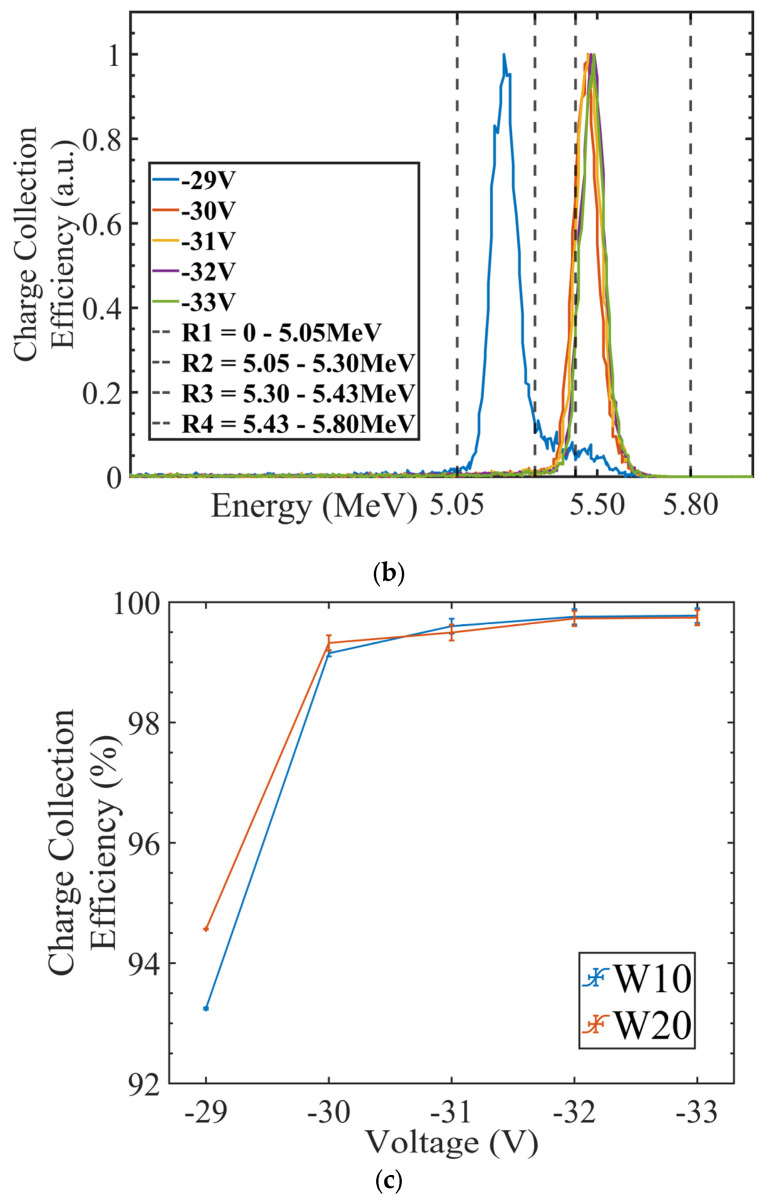
(**a**) Wafer10 energy spectra; (**b**) Wafer20 energy spectra; (**c**) charge collection efficiency (CCE) as a function of the bias for W10 and W20 wafers with 100 and 48 micron substrate thicknesses, respectively. Error bars indicate one standard deviation from the mean value of the alpha peak energy.

**Figure 8:**
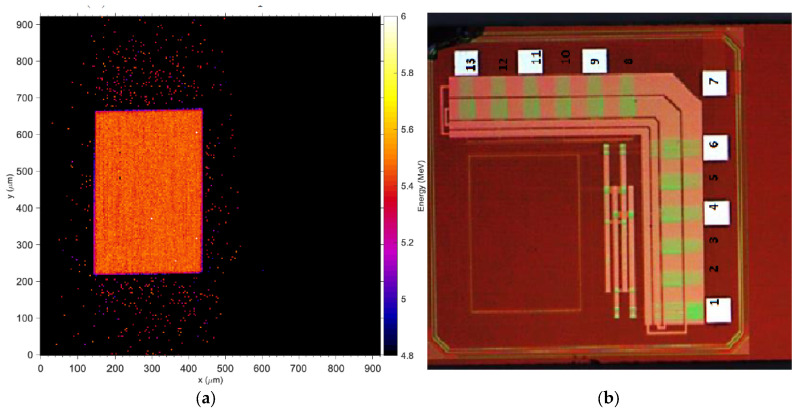
Direct comparison of charge collection map at −31 V (**a**) and microphotography of the layout of the sensor. The faint light red mark in (**b**) shows the actual sensitive area of the detector.

**Figure 9:**
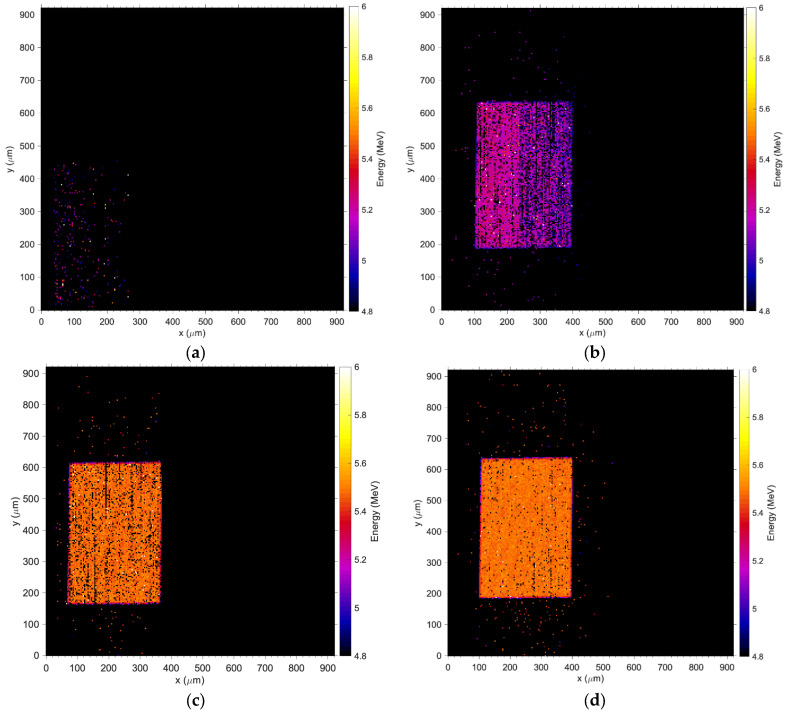
Wafer10 Median Energy Maps; (**a**) −28 V, (**b**) −29 V, (**c**) −31 V and (**d**) −32 V. The coordinates in x and y are obtained using a calibration factor that converts the electric potentials applied to the steering magnets of the accelerator to a physical distance of the spot at the plane where the device under test is positioned, please refer to [39] for details on the calibration procedure adopted at ANSTO. The coordinate frames of the pictures are consistent between the figures but they can be obtained using a different offset, which results in a shift of the map relative to the axis.

Figure 7a,b depict the measured energy spectrum for the 5.5 MeV alpha particles detected by Wafer10 and Wafer20 detectors when a bias up to −33 V is applied. The normalized energy obtained from the induced (collected) charge saturates as the bias voltage increases (Figure 7c). The spectra shown in Figure 7a,b and the plot in Figure 7c confirm the results obtained with the IV measurements, where a minimum of −31 V is required for full charge collection. Figure 8 shows the Median Map at −31 V with a total exposure time of 5 min compared to an optical picture of the detector. The sensitive volume corresponding to the pad detector shows an average count rate up to 900 events/second (maximum beam flux over 1 × 1 mm^2^) while the regions associated with the electronics show only few events per second associated with shot noise from cables and connections. The Median Maps in Figure 9 integrate all of the events in the spectrum and show that any bias below −29 V produces a partial charge collection but also generates a gradient of the electric field across the diode (Figure 9a,b). The spectra associated with these maps are shown in Figure 7a,b.

**Figure 10:**
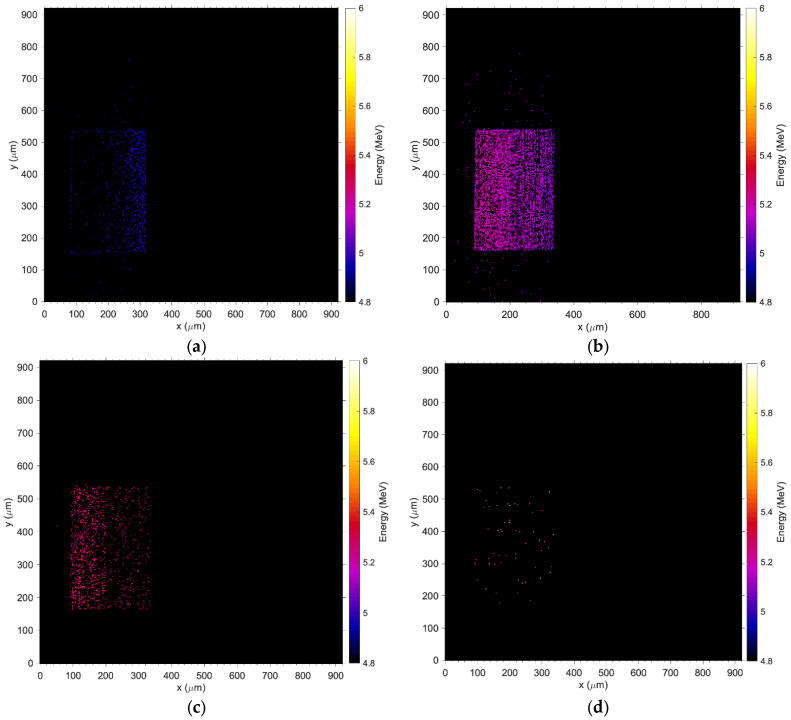
Median energy maps obtained at a bias of −29 V: (**a**) window set between 0 and 5 MeV; (**b**) when the window is set between 5–5.1 MeV, (**c**) between 5.1–5.235 MeV, (**d**) and between 5.235–5.80 MeV.

**Figure 11:**
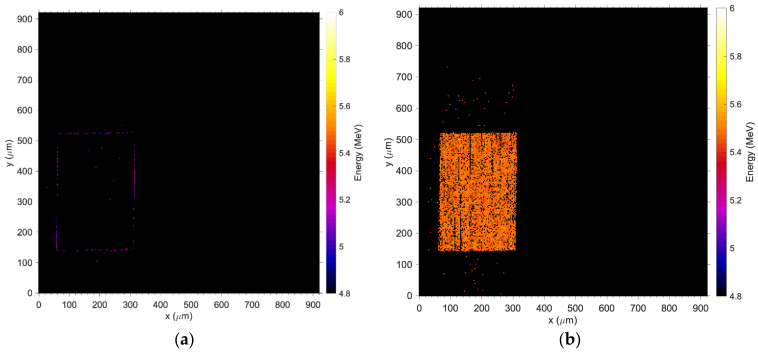
Median energy map when the sensor is biased at −31 V; In the energy window between 0–5 MeV, no events are registered in the area of the sensor nor in the area dedicated to the electronics; (**a**) is the map of the events registered in the window from 5 to 5.235 MeV and (**b**) events between 5.235 and 5.60 MeV.

In contrast to Figure 9a,b, the medium energy maps in Figure 9c,d show that charge collection is fully restored at −31 V and the efficacy of the guard ring, which effectively shields the pad from partially recombined events occurring outside the principal sensitive volume. Figure 10 shows the median energy windowed maps (MEWM) of the charge collection with a map of the events correlated to their energy. In Figure 7 and Figure 10a,b, the guard ring is not fully effective since the bias applied to the detector is below −31 V and the depletion region growing from the bottom of the substrate has not yet reached the top junction (Figure 11a,b). When the device is not fully depleted, minority carriers produced in the diffusion region can reach the main drift region where they contribute to the induced current. The low energy shoulder of the spectrum is due to the carriers that don’t reach the drift region and contribute to the induced (collected) charge.

### 3.2. Sensitivity and Calibration with Ir-192

**Figure 12:**
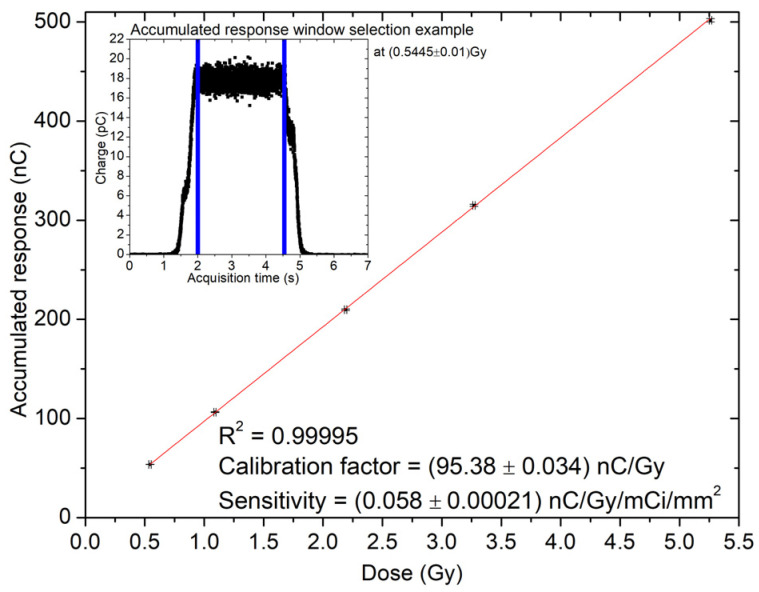
Variation of the response as a function of the accumulated dose for a bias voltage of −31 V. The symbol in the plot contains the error bars calculated as 1 standard deviation; the red line represents the linear fit used to calculate the calibration factor.

Table 1 shows the response of the sensor in nC as a function of the nominal dose calculated by the TPS for each dwell time and delivered to the detector position. Figure 12 shows the linear trend of the response with a coefficient of determination R^2^ > 0.99. The linear fit is used to calculate the sensitivity and calibration factor in nC/Gy.

Table 2 summarises the calibration factors normalised to the area of the detector and the activity of the source. Samples fabricated in Wafer10, with a substrate of 100 μm, show a sensitivity larger than the samples on Wafer20 (48 μm thick) due to its thicker sensitive layer of the substrate.

### 3.3. High Dose Rate (HDR) Source Localization with Ir-192

**Figure 13:**
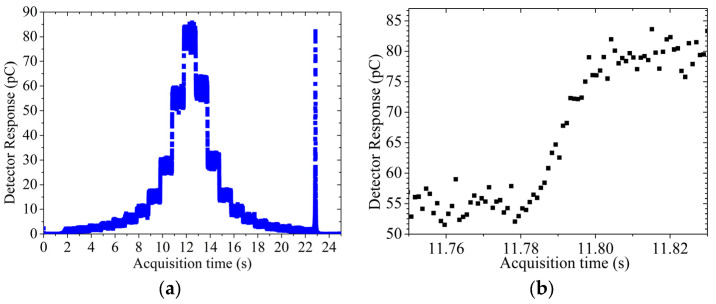
(**a**) data from source travelling at 2 mm depth with baseline subtracted, (**b**) transient of the source travelling 3 mm away from the detector.

Measurements were carried out with the sensor biased at −31 V for a total acquisition time of approximately 25 s, at 1 kHz sampling rate. Figure 13a shows the response, as a function of time, collected with the source dwelling at 2 mm distance above the sensor. It is apparent that the response remains constant during the dwell time (approx. 1 s) since the source is static in a dwell position. The transient in Figure 13b instead is the zoom-in of Figure 13a and represents the transient time between two dwell positions. Data displayed in Figure 13b are median values over 20 data samples to facilitate the visualisation of the data and provide error bars calculated as one standard deviation over three repetitions at the same measurement settings. The response in Figure 13a shows clearly the HDR source approaching the detector horizontally: the first dwell position is approximately 30 mm away from the sensor, then the source stays stationary for 1 s and moves to the next dwell position in increments of 3 mm.

The source reaches the minimum distance of 2 mm above the sensor at approximately 13 s and then travels along the catheter until reaching its tip. When the source reaches the tip of the catheter, the source is retracted at maximum speed into the afterloader generating a large pulse in the sensor as recorded at 23 s in the acquisition. This pulse of response corresponds to a negligible contribution of dose but allows the calculation of the maximum transient velocity of the afterloader. The velocity of 32.97 ± 4.80 cm/s is typical of the maximum velocity for a modern afterloader. A similar result for a Flexitron HDR afterloader, measuring approximately 35 cm/s, was obtained in [11].

**Figure 14:**
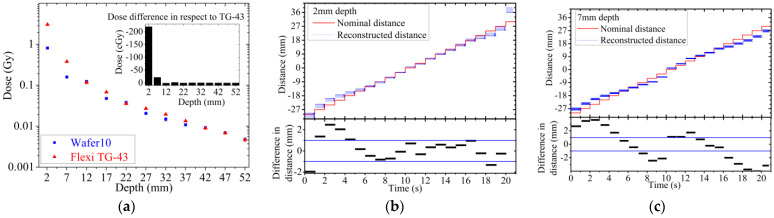
(**a**) Dose difference respective to TG-43. (**b**) Reconstructed distance of the source travelling along the catheter placed at 2 mm and (**c**) 7 mm above the source compared to the TPS nominal plan.

The depth dose curve for the sample fabricated on Wafer10 (Figure 14a) is derived using the response of the sensor in pC and applying the calibration factor calculated from sensitivity measurement (Figure 12). The depth dose data are compared to TG-43 values to benchmark the detector precision in estimating the actual absorbed dose delivered by the source in a water equivalent phantom. For depths larger than 12 mm, agreement of the data is within +/−0.2 cGy, which corresponds to a discrepancy within 5%. On the other hand, large discrepancies are observed for distances between 2 and 7 mm. The strong under-responsiveness obtained at shallower depths is due to the effects of scattering and radiation field perturbation created by the ceramic/golden package adopted for assembling the sensor prototypes. The ceramic package also creates a large air gap (few mm^3^) around the sensor due to the bonding cavity (10 × 10 × 2 mm^3^). The airgap combined with the ceramic walls of the cavity perturbs the flux of secondary electrons in close proximity of the detector, reducing the dose recorded by the sensor in respect to the dose calculated by the TPS. This result is certainly sub-optimal and will require further improvement of the package in order to match the requirement of dose measurement with a maximum discrepancy of 5% from the TPS for distance above 5 mm from the source. The impact of the package adopted is confirmed by the results shown in Figure 14b,c. The data represent the comparison between the distance of the source as a function of time as planned by the TPS and as reconstructed from the response of the detector. The data show a significant angular dependence in the ability to reconstruct accurately the position of the source along the catheter. Positions within a range of ±21 mm from the central position (corresponding to position at dwell interval of t = 10 s) are reconstructed with an accuracy of ±1 mm in respect to TPS (Figure 14b) if the catheter is at 2 mm depth. Positions at larger distances and wide angles have more significant discrepancies from the expected values; these positions appear systematically closer than expected with discrepancies within ±2 mm (Figure 14c). The ceramic cavity acts as a layer of material with high attenuation of the photons and secondary electrons. This condition is pronounced when the source is dwell in positions further away from the sensor. The geometry of the setup is described in detail in Figure 3 and shows a clear correlation between position of the source and under-estimation of the reconstructed position based on the sensor response.

**Figure 15:**
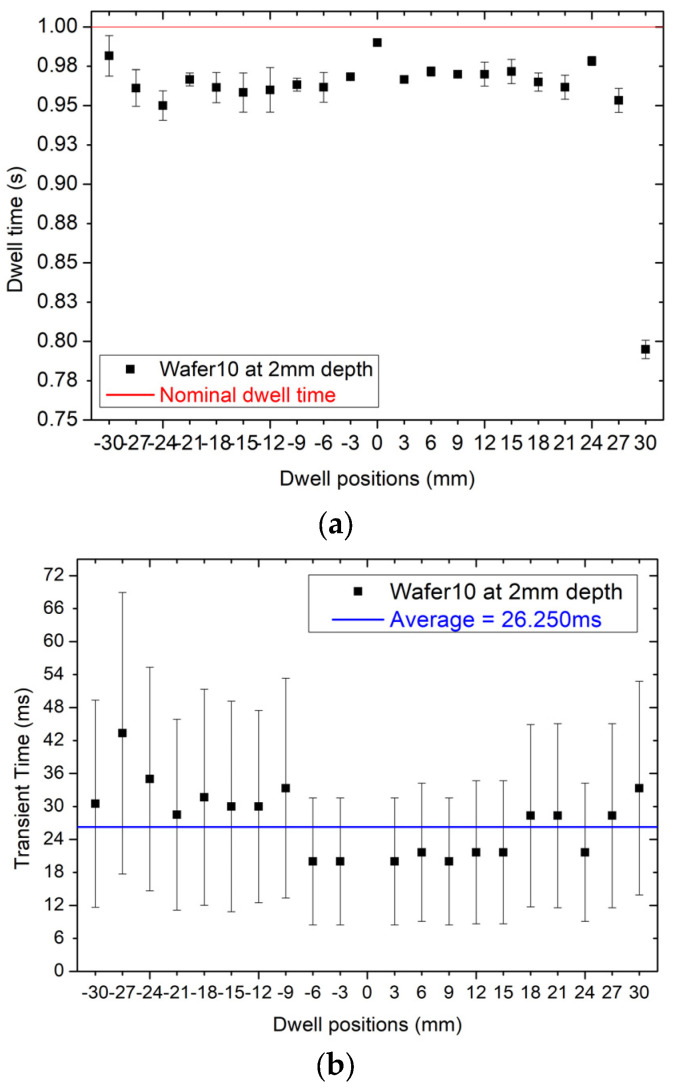
(**a**) dwell time at different dwell positions at 2 mm depth, (**b**) transient time at different dwell position at 2 mm depth.

Figure 15a shows the reconstruction of the dwell time for each dwell position. On average, the dwell time is between 20 to 30 ms shorter than the nominal dwell time of 1 s. This offset is not a discrepancy created by the sensor, but it is a feature of the afterloader that sets the dwell time always shorter than the nominal time to take into account the extra dose delivered by the source during the transient time between dwell positions. The transient time depends on the distance between dwell positions so the value of the dwell time is scaled accordingly. The afterloader applies this offset automatically and its value can be assessed only experimentally. The average discrepancy between the nominal dwell time of the TPS of 1 s in respect to the sum of the transit time to dwell time for each dwell position is 7 ± 2 ms corresponding to 0.7%.

The fast responsiveness of the detector allows for an independent verification of this feature by sampling the response at a frequency of 1 kHz to reconstruct the transient of the dose between dwell positions.

Figure 15b shows the transit time of the source between dwell positions (21 dwell positions for the specific plan adopted) which can be extracted by calculating the interval of time between 20% and 80% of the difference between the initial and final dwell position responses, respectively. Figure 15b confirms that the variation of the transient time as a function of the dwell positions is on average approximately 26 ms as predicted by the measurement of the dwell time. Error bars in Figure 15b show a fluctuation about the average value of the transit time of approximately ±20 ms, which is reported also in [11] and explained as an effect generated by the combination of the reproducibility of the sensor and the afterloader in running the source between dwell positions.

## 4. Discussion and Conclusions

This work presents the comprehensive characterization of an active CMOS pad sensor proposed for dosimetry and tracking in HDR brachytherapy. The sensor is a high resistivity silicon detector built on a standard CMOS substrate along with two amplifiers: a transimpedance amplifier and a charge sensitive amplifier. The former configuration has been used for this study and two geometries of the detector has been investigated: Wafer10 fabricated on a 100 μm thick substrate and the Wafer20 fabricated on a 48 μm epitaxial substrate. Basic current-voltage characteristics were measured to establish the optimal bias of the sensors. Full depletion is achieved for Wafer10 and Wafer20 at −31 ± 0.1 V and 30 ± 0.1 V, respectively. Full charge collection in tested detector is confirmed by the energy spectra obtained from IBIC microscopy measurements. The IBIC microscopy also confirmed the absence of parasitic charge collection due to direct interaction of radiation with the CMOS electronic stages.

The characterization of the response of the sensor exposed to an Ir-192 gamma source for HDR brachytherapy has been characterized for both tracking of the source and for dosimetry in a point in a water equivalent phantom. Tracking is very sensitive to variation of the density of the phantom and results showed an under-estimation of the reconstructed distance of the source for large angles while for narrow angles corresponding to the source directly above the sensor, the accuracy in reconstruction was within ±1 mm. The data acquisition system adopted for the experiment and the fast response of the embedded electronic amplifier allowed for accurate estimation of the dwell and transient time but it was a sub-optimal solution. In the future, a dedicated DAQ will be developed, with the capability to have multiple channels and direct digital conversion of the voltage output signal of the sensor without the need of the resistor R2 used in the original configuration. The sensor measured the dwell time delivered by the afterloader with an accuracy within 7 ms for all dwell positions defined in the treatment plan taking into account the effect of the transient time. The sensor has been also calibrated in dose and sensitivity recorded as (5.8 ± 0.021) × 10^−8^ nC Gy^−1^ mCi^−1^ mm^−2^ and (3.6 ± 0.14) × 10^−8^ nC Gy^−1^ mCi^−1^ mm^−2^ for Wafer10 and Wafer20, respectively. The sensitivity scales non-linearly with the substrate thickness due to the soft spectrum of the source. After calibration, a depth dose curve has been measured by dwelling the source above the sensor interposing slabs of solid water to increase the distance between them. PDD shows an agreement within 1 cGy for depths from 12 to 55 mm. Large discrepancies are recorded between the data of the TPS and the sensor for shallower depths due to the excessive radiation field perturbation created by the sub-optimal packaging of the samples. When the radiation source is in very close proximity of the sensor the spectrum is dominated by low energy photons and the excess of ceramic and golden plated extra-cameral materials enhances the absorption and perturbation of the flux of particles. This effect is mitigated at larger depth due to the filtration generated by the water equivalent material of the phantom and the larger spread of the photons and electrons generated by scattering.

Several technological solutions are available to produce a package for the sensor, which will minimise the angular dependence of the response of the sensor, but further studies are required to evaluate their impact on the radiation field perturbation and mechanical robustness.

The first prototype of this innovative dosimeter developed for HDR brachytherapy, adopting a standard CMOS silicon technology, is promising since it provides a fast, low noise and reliable detector with capability of tracking and dosimetry with a very simple readout data acquisition system and virtually no need for noise rejection or complex shielding. This technology allows the implementation of multipixel arrays for HDR quality assurance such as a probe compatible with a French catheter for the urethra or embedded in a sleeve for rectum wall multi point dosimetry or tracking.

## Figures and Tables

**Table 1:** Measured charge as a function of delivered dose.

Dwell Time (s)	Dose (Gy)	Integral Response (nC)
3	0.54 ± 0.01	53.69 ± 1.00
6	1.09 ± 0.01	106.37 ± 1.04
12	2.18 ± 0.01	209.86 ± 1.21
18	3.27 ± 0.01	315.09 ± 1.48
29	5.26 ± 0.01	501.81 ± 2.02

**Table 2:** Wafer10 and Wafer20 sensitivity.

Wafer	Sensitivity (nC Gy^−1^ mCi^−1^ mm^−2^)
W10	(58 ± 0.21) × 10^−3^
W20	(36 ± 1.4) × 10^−3^

## Data Availability

Data is unavailable due to IP restrictions.
